# Prognostic significance of midwall fibrosis in dilated cardiomyopathy

**DOI:** 10.1186/1532-429X-14-S1-O6

**Published:** 2012-02-01

**Authors:** Ankur Gulati, Ravi Assomull, Kishen Morarji, Tristan Brown, Emmanouil Liodakis, Marc R Dweck, Zohya Khalique, Jiten Morarji, Francisco Alpendurada, Sanjay K Prasad

**Affiliations:** 1 Royal Brompton Hospital, London, UK

## Background

Small studies with composite endpoints show that midwall fibrosis, identified by late gadolinium enhancement cardiovascular magnetic resonance (LGE-CMR), predicts short term adverse prognosis in dilated cardiomyopathy (DCM). We hypothesized that midwall fibrosis is an independent predictor of mortality over a long follow-up period.

## Methods

Consecutive patients with DCM referred for CMR between 2002-2008 were prospectively enrolled. The diagnosis of DCM was made using clinical, CMR and coronary angiographic findings. Patients with ischemic heart disease, primary valvar disease and infiltrative CM were excluded. LGE-CMR at 1.5T (Siemens Sonata or Avanto, Germany) was performed in 2 phase-encoding directions. The presence of midwall LGE was determined by a specialist blinded to all outcome data. The primary endpoint was all-cause mortality. The secondary endpoint was a composite of cardiovascular (CV) mortality or cardiac transplantation.

## Results

**Figure 1:**
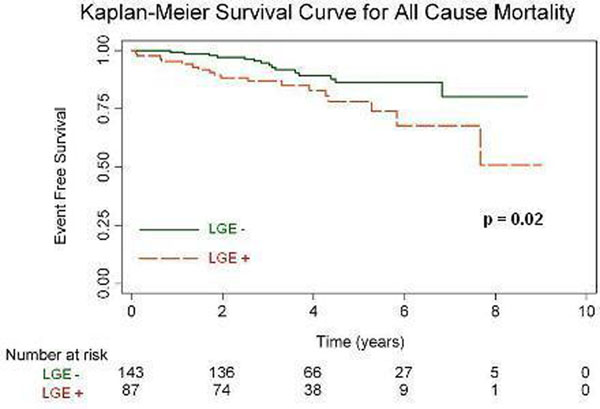
Kaplan-Meier survival estimates for all-cause mortality. Patients are grouped by presence (LGE+) or absence (LGE-) of midwall LGE.

Two hundred and thirty patients (157 male, mean age 50 yrs, mean LVEF 35%) were followed up for a median duration of 44 months. Midwall fibrosis was identified in 87 (38%) patients. No patient was lost to follow-up. There were 33 deaths (26 CV deaths, 7 non-cardiac) and 7 patients underwent cardiac transplantation. Kaplan-Meier analysis demonstrated a significant association between the presence of midwall fibrosis and both the primary (p=0.02, Fig.1) and secondary (p=0.003) endpoints. Midwall fibrosis remained a significant independent predictor of all-cause mortality (HR 2.2, 95% CI 1.1 to 4.7; p=0.035) and CV mortality/transplantation (HR 2.8, 95% CI 1.3 to 6.0; p=0.007) on stepwise Cox regression multivariable analysis allowing for conventional outcome predictors.

## Conclusions

Midwall fibrosis is a significant independent predictor of both all-cause mortality and CV mortality/transplantation in DCM. Detection of midwall fibrosis by LGE-CMR represents an important and novel marker for the risk stratification of DCM patients.

## Funding

This work was supported by the NIHR Cardiovascular Biomedical Research Unit at the Royal Brompton and Harefield NHS Foundation Trust and Imperial College London. Dr Ankur Gulati receives salary support from CORDA.

